# Targeting Caspase 8: Using Structural and Ligand-Based Approaches to Identify Potential Leads for the Treatment of Multi-Neurodegenerative Diseases

**DOI:** 10.3390/molecules24091827

**Published:** 2019-05-12

**Authors:** Khurshid Ahmad, Vishal M. Balaramnavar, Navaneet Chaturvedi, Saif Khan, Shafiul Haque, Yong-Ho Lee, Inho Choi

**Affiliations:** 1 Department of Medical Biotechnology, Yeungnam University, Gyeongsan 38541, Korea; ahmadkhursheed2008@gmail.com; 2 Department of Pharmaceutical Chemistry, GIPER, Kashipur, Uttarakhand 44713, India; v.balaramnavar@gmail.com; 3 University of Information Science and Technology, St. Paul The Apostle, Ohrid 6000, Macedonia; 14.navneet@gmail.com; 4 College of Dentistry, Hail University, Hail 2440, Saudi Arabia; Saifkhan.bio@gmail.com; 5 Research and Scientific Studies Unit, College of Nursing and Allied Health Sciences, Jazan University, Jazan 45142, Saudi Arabia; shafiul.haque@hotmail.com; 6 Department of Biomedical Sciences, Daegu Catholic University, Gyeongsan 38430, Korea

**Keywords:** caspase 8, ligand, pharmacophore, neurodegeneration, virtual screening

## Abstract

Caspase 8 is a central player in the apoptotic cell death pathway and is also essential for cytokine processing. The critical role of this protease in cell death pathways has generated research interest because its activation has also been linked with neural cell death. Thus, blocking the activity of caspase 8 is considered a potential therapy for neurodegenerative diseases. To extend the repertoire of caspase 8 inhibitors, we employed several computational approaches to identify potential caspase 8 inhibitors. Based on the structural information of reported inhibitors, we designed several individual and consensus pharmacophore models and then screened the ZINC database, which contains 105,480 compounds. Screening generated 5332 candidates, but after applying stringent criteria only two candidate compounds, ZINC19370490 and ZINC04534268, were evaluated by molecular dynamics simulations and subjected to Molecular Mechanics/Poisson Boltzmann Surface Area (MM-PBSA) analysis. These compounds were stable throughout simulations and interacted with targeted protein by forming hydrogen and van der Waal bonds. MM-PBSA analysis showed that these compounds were comparable or better than reported caspase 8 inhibitors. Furthermore, their physical properties were found to be acceptable, and they are non-toxic according to the ADMET online server. We suggest that the inhibitory efficacies of ZINC19370490 and ZINC04534268 be subjected to experimental validation.

## 1. Introduction

Neurological disorders (NDs) are usually due to structural and/or functional losses of neurons and eventually neuronal death [1]. Literally, hundreds of neurological/neurodegenerative diseases have been identified, but some like Alzheimer’s disease (AD) and Parkinson’s disease (PD) are very well known and have devastating impacts in modern society. NDs tend to share some characteristics such as memory loss and the aggregation of abnormal proteins [2]. Neuronal death is the main characteristic of major NDs, and apoptosis is considered a possible mechanism of neuronal death in the majority of NDs [3]. Furthermore, abnormal or excessive neuron apoptosis eventually lead to a number of incurable diseases including AD, PD, Huntington’s disease (HD), and stroke [4,5]. The genes that crucially contribute to AD (β-amyloid precursor protein (APP), and presenilin-1 and -2) have been demonstrated to regulate apoptosis, which intimates dysregulation of apoptosis plays a notable role in triggering the neuronal loss in AD [6].

Caspases are the main executioners of apoptosis and are usually proteolytic in nature [7]. Caspases constitute a distinctive set of proteases (cysteine aspartate-specific) with the specific substrate and biological functions [8,9]. They are categorized as ‘inflammatory’ or ‘apoptotic’ on the basis of their functions and pro-domain structures and may further be categorized as initiator (caspase 2, 8, 9, and 10) or effector caspases (caspase 3, 6, and caspase 7) [10,11]. Caspases have been identified in the brains of AD patients, and caspases 1, 2, 3, 5, 6, 7, 8 and 9 have all been reported to be transcriptionally up-regulated in AD [12]. Several studies, including neuropathologic and clinical imaging observations, indicate that stimulated microglia (the monocyte-derived macrophage-like resident immune cells of the central nervous system) are primarily responsible for the pathogeneses of a number of NDs, including AD, PD, and multiple sclerosis [13,14,15]. Activated microglia release neurotoxic pro-inflammatory factors [16], and accumulated evidence indicates caspases (especially, caspase 3/7 and caspase 8) are key regulators of microglial activation [14,17]. Furthermore, neuroinflammation in the brains of AD and PD patients is attributed to the presence of activated microglia [15,17]. Thus, it has been suggested the identification of potent caspase inhibitors might prove to be a potent strategy for identifying neuroprotective agents for the treatment of several NDs [18,19].

Caspase 8 is involved in apoptosis and cytokine processing. The latter represents an initial step of the apoptotic cascade, which initiates proteolytic stimulation of downstream caspases and proceeds to apoptosis [20]. Activated caspase 8 has been detected in the AD brain, and its activation has been posited to be due to the stimulation of receptors in the ‘death-receptor pathway’ [21]. Furthermore, the stimulation of caspase 8 in activated microglia prevents their committing to necroptosis. Consequently, the identification of effective caspase 8 inhibitors might protect neurons by selectively killing or blocking the activities of activated microglia [22,23].

Activated caspase 8 has been detected within insoluble elements in HD brains [24], and reported to be significantly up-regulated in cerebrospinal fluid in amyotrophic lateral sclerosis (ALS) [25]. In addition, activated caspase 8 levels are significantly elevated in patients suffering from Dentatorubralpallidoluysian atrophy (DRPLA), a rare ND with a genetic pathology of polyglutamine (CAG) repeats [23,24]. In this study, we applied state of art in silico approaches, that is, pharmacophore modeling, virtual screening, molecular dynamics, molecular docking simulations, and MM-PBSA analysis in an attempt to discover potential caspase 8 inhibitors.

## 2. Results and Discussion

**Figure 1:**
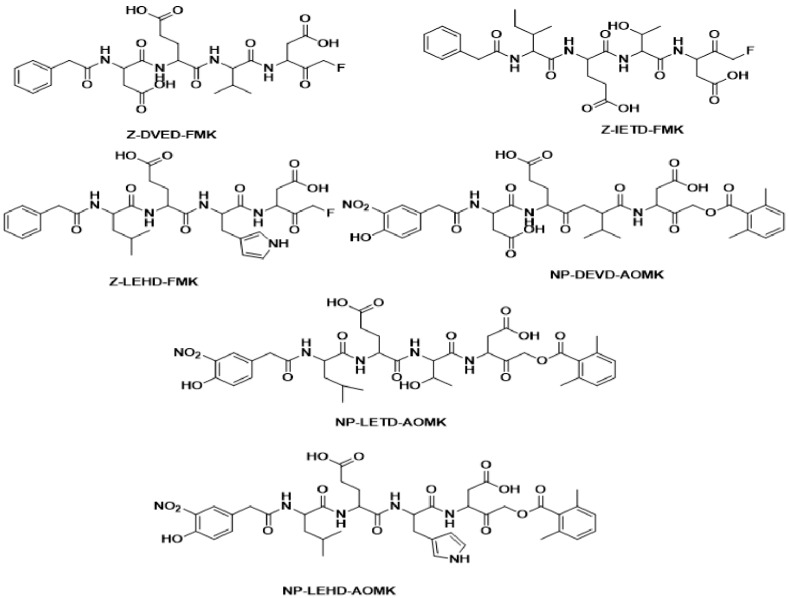
Structures of the training set compounds used for pharmacophore generation [26].

Due to the small activity range differences between training set compounds, we used the HipHop protocol for pharmacophore generation. We assumed that the most active ligand(s) in the training set would bind in the same manner with the active site of caspase 8. We assessed the common features essential for binding using the HipHop module in Catalyst software. The six-molecule training set was used to generate pharmacophore models based on common chemical features (Figure 1).

### 2.1. Pharmacophore Modeling: 3D Pharmacophore Generation

**Figure 2:**
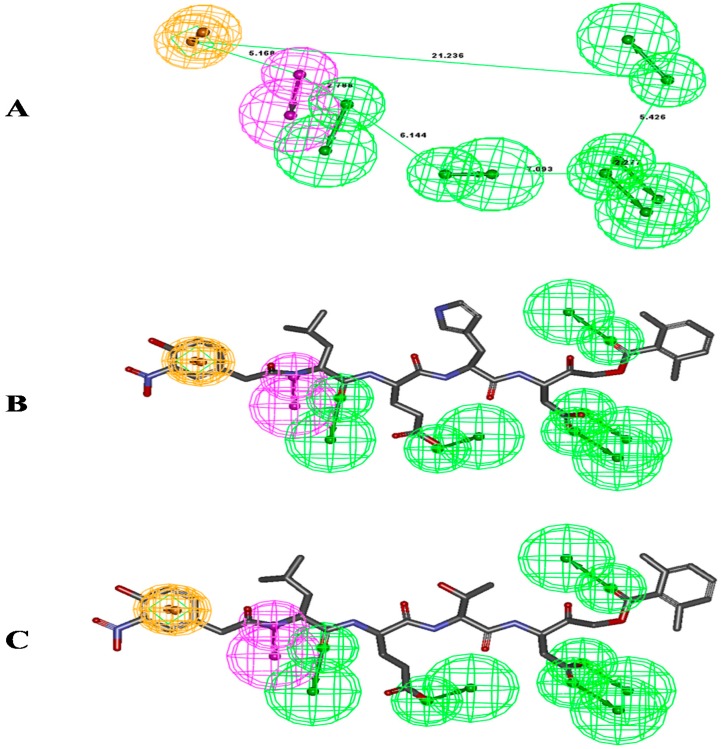
Pharmacophore mapping of compounds (**A**) Pharmacophore model developed using the training set compounds. (**B**) Mapping of the most active compound NP-LHED-AOMK from the training set compounds. (**C**) Pharmacophore mapping of the least active compound NP-LTED-AMPK on pharmacophore. The color represents R, ring aromatic (Orange); D, hydrogen bond donor (Purple); A, hydrogen bond acceptor; H (Green), hydrophobic group and P, positive ionisable (Red).

Ten pharmacophore models (hypotheses) were generated with a rank score ranging from 152.441 to 150.863 (Table 1). The resulting 10 hypotheses contained seven features viz. five hydrogen bond acceptor-lipid (H), one ring aromatic (R), and one hydrophobic (Z) common to all 10 hypotheses. Of these 10 hypotheses, Hypo-1 (Figure 2A) was selected as it mapped all features of the most active molecule in the six-compound training set. Two out of five H functions maps on the five oxygen with different –OH and C=O functions of the most active compound (NP-LHED-AOMK) (Figure 2B) from the training set, while the R function was mapped to one of the ring aromatic and hydrogen bond donor functions. The NH group from the amino acid linkage supported the remaining single hydrogen donor functionality. One benzene ring from LHED and LTED was mapped for the R function of this pharmacophore model (Figure 2C).

In the direct hit mask, (1) every feature of the training set molecules were mapped; (0) indicates one or more features were not mapped. In the partial hit mask, (0) indicates every feature of the training set molecules were mapped; (1) indicates one or more features were not mapped. Z; Hydrophobic (Z), H; Hydrogen bond acceptor (H), R; Ring aromatic (R).

### 2.2. Pharmacophore-Based Virtual Screening (PBVS)

The validated hypothesis (pharmacophore model) Hypo-1, was used as a query to screen the ZINC database, which contained 105,480 molecules, using the ‘Best Flexible Search’ option in DS 2.5. A hit list of 5332 compounds matched the query (Hypo1). Further filtration based on fit values of >3.5 and physiochemical screening resulted in 76 hits. These hits were subjected to visual inspection for proper alignments with Hypo-1, and finally, after performing molecular docking, 10 hits were retrieved from the ZINC database (Table 2). The final selection from this set of 10 hits was made using Moldock and Re-rank scores and resulted in the selection of two compounds (ZINC19370490 and ZINC04534268) for further study.

### 2.3. Molecular Dynamics Simulation and Molecular Docking

Molecular dynamics simulation of caspase 8 was performed for 20 ns using GROMOS96 53a6 force field. We analyzed the binding efficiencies of ZINC19370490 and ZINC04534268 with caspase 8 for different times (0 ns, 5 ns, 10 ns, 15 ns, and 20 ns). These selected compounds were further tested to confirm their binding affinities. A different scoring function (the gold fitness score) was used to reconfirm their binding affinities with caspase 8. This function scores the binding efficacies of receptor-ligand complexes, and in the present study were used to predict binding affinity [27,28]. The binding efficiencies of both compounds were determined and additional poses were created based on gold fitness scores. The binding efficacies of ZINC19370490 and ZINC04534268 with caspase 8 at different time intervals are shown in Table 3 and Table 4. 

**Figure 3:**
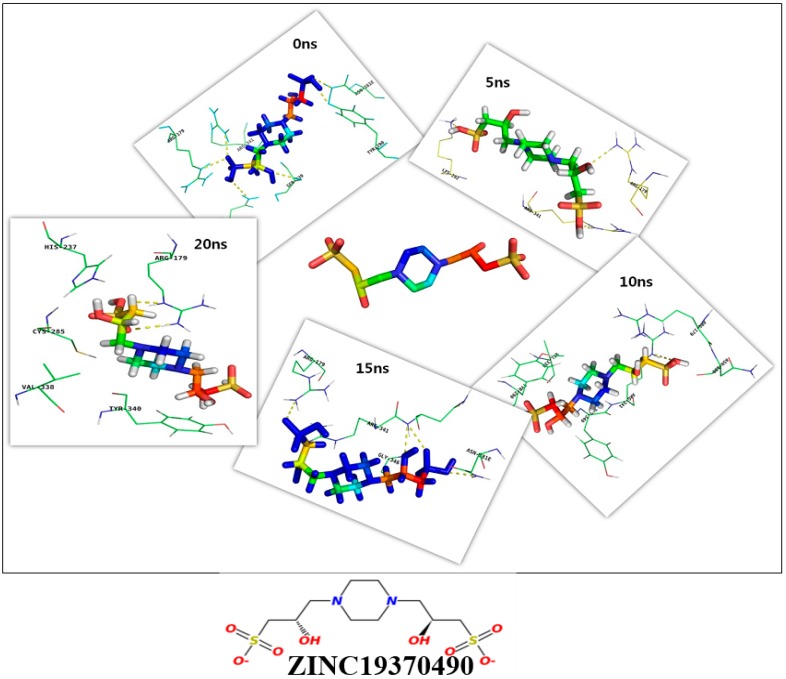
Binding mode of ZINC19370490 with active site residues of caspase 8 at different time intervals.

**Figure 4:**
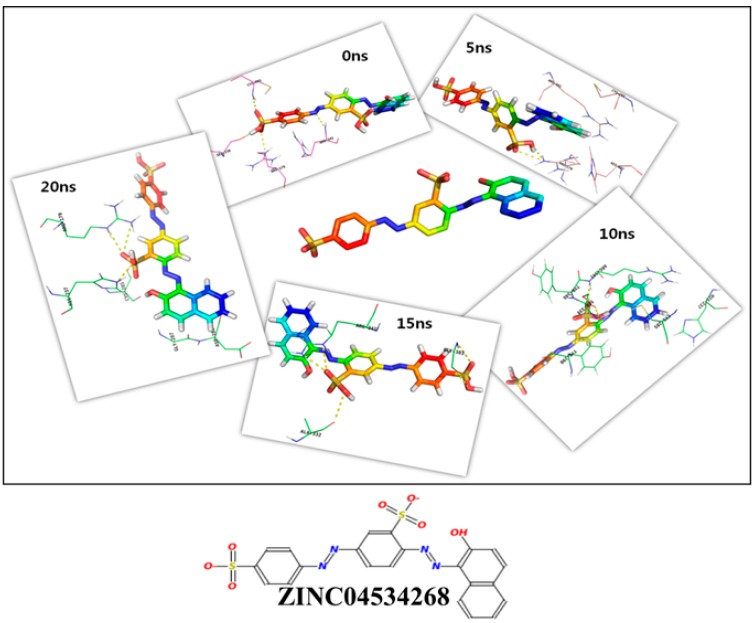
Binding mode of ZINC04534268 with active site residues of caspase 8 at different time intervals.

We found both compounds bound effectively to the active site of caspase 8. The detailed binding mechanisms of the two compounds were further investigated by analyzing binding poses using PyMOL [29]. Figure 3 and Figure 4 depict binding between ZINC19370490 or ZINC04534268 and the active site of caspase 8 at 0 ns, 5 ns, 10 ns, 15 ns, and 20 ns. R179, Y340, R341 and C285 residues in the active site of caspase 8 were found to be most involved in the binding of both compounds. The roles of these key residues have been also described elsewhere [30], and concur with our finding regarding the importance of these residues in the active site. Roles of other important amino acids were also revealed by the present study (Table 3 and Table 4). Summarizing, we found caspase 8 tends to form stable complexes with ZINC19370490 and ZINC04534268.

### 2.4. Thermodynamics Analysis

Thermodynamic analysis produced encouraging results, as binding free energies of ZINC19370490 and ZINC04534268 were either comparable or better than those of previously reported caspase 8 ligands [26] (Table 5). Thermodynamic parameters are important for drug design as they determine the relative binding strengths of protein and non-protein entities. Our MM-PBSA analysis showed the values of different parameters for ZINC19370490 and ZINC04534268 were comparable to those of existing molecules. In addition, we analyzed the solvation free energies of the two proteins to evaluate their water binding affinities, which is a critical consideration when designing molecular therapies. Interestingly, this analysis revealed ligand-binding sites exhibited both positive and negative solvation free energy regions/residues. The number of residues that avoid solvation is in general higher as compared to those that prefer to be soluble. The ligand-binding site of caspase 8 is surrounded by polar residues that pose a barrier to ligand binding.

### 2.5. Physical Properties of Ligands

The physical properties of these ZINC19370490 and ZINC04534268 were found to be compatible with the implementation of pre-clinical studies (Table 6). Lack of cytochrome P family inhibition is a good indicator of a long circulation half-life, low skin permeability reduces the risk of skin-related injuries, and low gut absorption can be enhanced by techniques such as nanoparticle encapsulation [31]. ZINC19370490 and ZINC04534268 are also non-toxic according to the ADMET online server (https://preadmet.bmdrc.kr/toxicity/).

## 3. Materials and Methods

### 3.1. Common Feature Pharmacophore Model

Based on our previous studies on approaches to indirect drug design [32,33] and in the field of neurodegenerative disease [33] we decided to focus on caspase 8 inhibitors. Molecules reported in the literature were collated [26], but biological activity data did not exhibit the required 3 log unit variation necessary to develop a quantitative pharmacophore model using the Hypogen module in Catalyst software. Accordingly, we built a common feature pharmacophore ‘HipHop’ model using six structurally diverse compounds with different inhibitory activities (training set, Figure 1).

### 3.2. Common Feature Pharmacophore Generation

The set of six diverse compounds with maximum activity at the dose of 250µm were used as the training set (Figure 1). Pharmacophore generation protocol was performed using the HipHop algorithm of Catalyst employed in Discovery Studio 2.0 (DS 2.5) [34]. All training set compounds were drawn/built using ISIS Draw 2.5 and imported into DS 2.5 windows. The CHARMm force field was applied to optimize training set compounds [35]. The conformations of these compounds were generated using the ‘diverse conformation generation’ protocol of DS 2.5 using default parameters (principal value = 2, maximum omit feature = 0). Accordingly, the most active compound was assigned a score of 1 and moderately and less active compounds were assigned a score of 0. The ‘Feature Mapping’ protocol was run to detect common features in the training set.

### 3.3. Pharmacophore-Based Virtual Screening (PBVS)

The PBVS approach was used to identify probable inhibitors of caspase 8. The validated model of pharmacophore (Hypo-1) was used as a query to search for compounds in the ZINC database using ‘Best flexible search’ option in DS 2.5. Resulting hits were screened based on fit values of >3.5 and this was followed by additional screening using physiochemical properties. In addition, these hits were subjected to visual inspection for proper alignments with Hypo-1 and finally subjected to molecular docking, after completing the virtual screening process the 10 most potent hits were retrieved from the ZINC database. Two of these 10 hits were selected based on Moldock score and re-rank scores for further study.

### 3.4. Molecular Docking Studies

Molecular docking was performed using the MolDock module in Molegro Virtual Docker (MVD) software [36]. The scoring function of molecular docking in MolDock is based on piecewise linear potentials (PLPs) [37]. A re-ranking method was applied to the highest ranked poses to increase the accuracy of docking. The search algorithm ‘MolDock SE’ was applied to this analysis, and population size of 50 and a maximum number of iterations of 1500 were set as parameters. Other parameters were kept as default with the number of runs as 10. Since MVD relies on an evolutionary algorithm, repeated docking runs do not result in precisely the same poses and interactions. To address this intrinsic arbitrariness, three successive runs were performed and the best three poses were used to visualize further interactions.

### 3.5. Molecular Dynamics Simulation

The Gromacs 4.6.7 suite was utilized to perform MD simulations [38] with the 53a6 parameter set of gromos96 force field [39]. A dodecahedron box was selected to immerse the protein and SPC216 water model was used to fill the box [40] that extended to at least 1.2 nm from the edge of the protein. Na^+^ and Cl^−^ counter ions were added to neutralize the system up to a physiological salt concentration of 100mM. After solvation and neutralization steps, system was minimized energetically using the steepest-descent method to remove steric clashes between atoms. In addition, the MD simulations were run under NPT (Constant number of particles, pressure and temperature) conditions with the use of Berendsen’s coupling algorithm for maintaining constant temperature and pressure. The LINCS algorithm [41] was used to constrain the lengths of all bonds and water molecules were restrained using the SETTLE algorithm [42]. The particle mesh Ewald (PME) was used to treat long-range electrostatic interaction [43]. A 10 Å cut-off was employed for the van der Waals interactions. The MD simulation time-step was 2 fs and coordinates were saved every 2ps. The PRODRG server was used for the parameterization and generation of topology for each ligand [44].

### 3.6. Thermodynamic Analysis

Protein-ligand binding free energies were calculated to monitor the binding intensities of ligands to caspase 8 using MM-PBSA. For the MM-PBSA calculations, the system was prepared by adding hydrogens, and partial charges on ligands were calculated using the theory utilized in the AM1-BCC model. Calculations were performed using AMBERTOOLS16. Solvation free energies and decomposition into individual amino acids were calculated using ProWave web server (https://www.prowave.org/). The physical properties of ligands were calculated using PreADMET [45] and SwissADME [46].

## 4. Conclusions

Common feature-based pharmacophore modelling and structure-based docking study were applied to identify novel caspase 8 inhibitors. A pharmacophore model (Hypo-1) was developed and validated and used as a filtering tool to screen the ZINC database. After a series of screenings followed by visual inspection and molecular docking of selected compounds, the two most potent compounds were subjected to molecular docking with caspase 8 at different time intervals (0 ns, 5 ns, 10 ns, 15 ns, and 20 ns) to obtain deeper insight of their binding efficacies.

The binding free energies of these two compounds were comparable or better than those of previously described caspase 8 inhibitors and satisfied most drug candidate criteria. Using a combination of pharmacophore modeling, virtual screening, molecular docking, and finally molecular dynamics simulations of these two proteins at different time intervals and their binding affinities, we conclude that ZINC19370490 and ZINC04534268 be viewed as potential lead compounds.

## Figures and Tables

**Table 1:** The characteristics of the generated pharmacophore model.

9	Features	Rank	Direct Hit	Partial Hit	Max Fit
**01**	RDHHHHH	152.441	111111	000000	7
**02**	RZHHHHH	152.286	111111	000000	7
**03**	RZHHHHH	152.086	111111	000000	7
**04**	RZHHHHH	152.058	111111	000000	7
**05**	RZHHHHH	151.770	111111	000000	7
**06**	RZHHHHH	151.719	111111	000000	7
**07**	RZHHHHH	151.511	111111	000000	7
**08**	RZHHHHH	151.147	111111	000000	7
**09**	RZHHHHH	151.017	111111	000000	7
**10**	RZHHHHH	150.863	111111	000000	7

(R, ring aromatic; D, hydrogen bond donor; H, hydrogen bond acceptor; Z, hydrophobic group and P, positive ionisable. Direct hit; all the features of the hypothesis are mapped. Direct hit = 1 means yes and Direct hit = 0 is no. Partial hit; partial mapping of the hypothesis. Partial hit = 1 means yes and Partial hit = 0 means no.).

**Table 2:** Functions resulted from docking of top 10 prioritized compounds.

Ligand	MolDock Score	Rerank Score	Docking Score	Similarity	Fit Value
ZINC00959167	−79.5965	−68.8523	−328.308	−247.343	4.999
ZINC01234548	−109.655	−94.2736	−362.447	−248.796	4.79982
ZINC09212678	−80.7085	−69.2156	−328.066	−246.039	4.89982
ZINC01301026	−72.0231	−58.011	−342.572	−265.101	4.99982
ZINC03830398	−76.8326	−60.6093	−345.951	−270.038	4.922
ZINC06143162	−88.6888	−65.8386	−339.951	−250.383	4.4982
ZINC14671560	−97.5435	−77.8008	−377.247	−280.961	4.482
ZINC04534268	−116.417	−94.1023	−440.055	−309.038	4.9482
ZINC19370490	−138.402	−117.46	−480.995	−329.518	4.682
ZINC02775438	−91.4008	−67.6985	−406.978	−311.933	4.621

**Table 3:** Efficacy of ZINC19370490 against caspase 8 at different time intervals and the active site residues involved in the binding.

Time Period	Gold Fitness Score	Residues	
		Hydrogen Bond	Hydrophobic Interaction
0 ns	53.78	R179, Q283, Y290, S339, R341, N381	R179, C285, S339, Y340, R341
5 ns	53.30	R179, K292, R341	R179, Y290, K292, Y340, R341, P343
10 ns	54.06	R341	R179, N180, Y290, V338, Y340, R341
15 ns	58.12	R179, R341, N342, G346, N381E	C285, T333, V338, S339, Y340, R341, E345, G346, T347, W348, N381E, Q385
20 ns	51.50	R179	H237, C285, V338, Y340

**Table 4:** Binding efficacy of ZINC04534268 against caspase 8 at different time intervals and the active site residues involved in the binding.

Time Period	Gold Fitness Score	Residues	
		Hydrogen Bond	Hydrophobic Interaction
0 ns	60.48	R179, S236, C285, R341	H237, C285, S339, Y340, R341, D381B, K381D,
5 ns	52.37	R179	R179, H237, C285, V338, R341, P343, N381E,
10 ns	55.96	S339, R341	R179, H237, C285, Y290, K292, S339, Y340, R341
15 ns	57.52	A332, R341, G383	R179, N180, G181, D185, Q283, Y340, R341, G346, T347, D381A, N381E, G383, Q385,
20 ns	58.68	R179, H237	S175J, H237, C285, G287, D288, Y290, V338, Y340, R341, P343,

**Table 5:** MM-PBSA values (binding free energies) of different ligands with caspase 8. Each value represents the average value calculated from the last 100 snapshots. SASA, solvent accessible surface area; SAV, solvent accessible volume. The standard deviations are given in parenthesis.

Ligand	Electrostatic	Polar Solvation	Van der Waal	SASA	SAV	Binding Free Energy (KJ/mol)
Z-IETD-FMK	−81.44 (33.4)	−4.98 (4.5)	−121.622 (15.2)	−14.52 (1.25)	−159.2 (9.5)	−382.524 (29.45)
Z-IETD-FMK	−155.45 (30.5)	58.326 (10.5)	−140.4 (8.45)	−17.90 (2.58)	−198.5 (6.47)	−453.924 (45.98)
Z-LEHD-FMK	−58.45 (21.25)	212.670 (12.36)	−108.45 (19.585)	−19.07 (2.04)	−169.7 (7.8)	−143.08 (29.704)
NP-DEVD_AOMK	−53.11 (48.90)	80.37 (4.80)	−109. 48 (5.08)	−13.09 (1.08)	−117.9 (6.24)	−106.99 (34.19)
NP-LETD-AOMK	−145.48 (46.2)	254.051 (15.66)	−314. 94 (18.56)	−17.6 (1.69)	−178.56 (9.4)	−401.93 (26.78)
NP-LEHD AOMK	−58.67 (45.09)	198.07 (12.360)	−265.98 (14.8)	−11.04 (2.05)	−164.5 (2.63)	−302.12 (41.29)
ZINC19370490	−154.45 (4.22)	109.45 (29.07)	−190.07 (26.6)	−19.05 (2.44)	−180.56 (4.5)	−434.68 (39.45)
ZINC04534268	−225.64 (79.82)	76.697 (9.14)	−217.56 (78.2)	−17.89 (1.99)	−88.67 (2.6)	−484.063 (31.08)

**Table 6:** General properties of ZINC04534268 and ZINC19370490.

Properties	ZINC04534268	ZINC19370490
GI absorption	Low	Low
BBB permeant	No	No
P-gp substrate	No	Yes
CYP1A2 inhibitor	No	No
CYP2C19 inhibitor	No	No
CYP2C9 inhibitor	No	No
CYP2D6 inhibitor	No	No
CYP3A4 inhibitor	No	No
LogK_p_(skin permeation)	−6.67 cm/s	−14.17 cm/s
